# Against the *De Minimis* Principle

**DOI:** 10.1111/risa.13445

**Published:** 2020-01-20

**Authors:** Björn Lundgren, H. Orri Stefánsson

**Affiliations:** ^1^ Institute for Futures Studies Stockholm Sweden; ^2^ Stockholm University Stockholm Sweden; ^3^ University of Twente Enschede The Netherlands; ^4^ Swedish Collegium for Advanced Study Uppsala Sweden

**Keywords:** *De minimis*, rational decision making, risk

## Abstract

According to the class of *de minimis* decision principles, risks can be ignored (or at least treated very differently from other risks) if the risk is sufficiently small. In this article, we argue that a *de minimis* threshold has no place in a normative theory of decision making, because the application of the principle will either recommend ignoring risks that should not be ignored (e.g., the sure death of a person) or it cannot be used by ordinary bounded and information‐constrained agents.

## 1 INTRODUCTION

According to the class of *de minimis* decision principles, a risk can be ignored (or at least treated very differently from other risks) if the risk is sufficiently small. There is some disagreement as to what makes a risk sufficiently small in this sense. According to Peterson (2002, p. 48), risks are *de minimis* if the probability that they materialize fall below some threshold (i.e., irrespective of the severity of that risk). In contrast, according to Fiksel (1985), a risk is *de minimis* if the expected value of the risk falls below some threshold. Sometimes, in order to simplify the argumentative structure, we stick closely to former formulation, but all our arguments hold, *mutatis mutandis*, for both formulations. A potential example of a *de minimis* risk, on either formulation, is health risks posed by cell phones’ electromagnetic fields (Peterson, 2002).

The principles within this *de minimis* class differ when it comes to how exactly a *de minimis* risk is treated; for instance, whether it should be completely ignored, or whether it triggers different risk mitigating strategies than other risks (for a discussion, see, e.g., Adler, 2007). Moreover, there is, among proponents of *de minimis*, vast disagreement on how this limit should be set (see Peterson, 2002).

The above differences and disagreements will (with one exception) not be relevant to our discussion. Instead, we focus on a general problem with giving risks below a threshold a special treatment. We shall argue that irrespective of how we set that threshold and whatever precise role the threshold plays, such a *de minimis* threshold has no place in a normative theory of decision making, that is, a theory about how one should choose.

In other words, rather than examining, say, actual risk analysis practices, to see whether a *de minimis* threshold is a useful component of such practices, we shall raise some theoretical problems that indicate that any risk analysis practice that includes such a threshold is deeply flawed, from a theoretical point of view. But we shall also illustrate through concrete examples how these theoretical problems can lead to great difficulties in practice.

It might nevertheless be argued that *de minimis* thresholds should be used as heuristics by boundedly rational agents—roughly, agents with limited cognitive abilities and imperfect information—who want to achieve results that come close to the results that they would achieve through more rational decision principles (see, e.g., Adler, 2007). To take an example, if it turns out that tiny probabilities of bad outcomes are sufficiently more expensive to eliminate than larger probabilities, then, as Mumpower (1986) points out, an application of a *de minimis* principle might lead to the same conclusion as an application of a full‐fledged cost‐benefit analysis (if the latter analysis could be carried out). However, we shall argue that since any acceptable application of *de minimis* should take into account the decisionmaker's total portfolio of risk, a *de minimis* principle will not be of much use to boundedly rational agents.

In developing our critique, we will rely on a previous discussion by Peterson (2002), in which three ways of setting the *de minimis* threshold are refuted in favor of a fourth option. We start by briefly describing and adding to Peterson's arguments, after which we turn to further specifying (or explicating) Peterson's own account. Next, we argue that proponents of the *de minimis* principle face a dilemma: Either it recommends treating as *de minimis* risks that should not be treated as *de minimis* (e.g., the sure death of a person) or it cannot be used by ordinary (bounded) agents. On this basis, we conclude that the principle should be dispensed with. We also add another argument against those formulations of *de minimis* that say that risks below some threshold can be *completely* ignored (as opposed to saying that such risks can be treated differently from other risks). We end the article with a brief concluding section.

It might be worth acknowledging right away that we will not directly engage with the positive arguments that have been made in favor of *de minimis* or similar principles.1We thank two anonymous reviewers for *Risk Analysis* for making us see the need to acknowledge the points raised in this paragraph and in the following final paragraphs of this introduction. For instance, some have argued that the correct response to games such as the St. Petersburg game and the Pasadena game, that have troubled decision theorists for centuries, is to ignore probabilities that fall below some threshold (for a recent argument along these lines, see Smith, 2014; for an overview, see Peterson, 2019, section 5). We ignore these arguments for two reasons. First, some of these arguments involve discussions about infinite sequences, which arguably are of little practical relevance. Second, our arguments aim to establish flaws with the *de minimis* principle, flaws so severe that we believe that they provide reasons to abolish the *de minimis* principle from rational decision making (at least those involving finite sequences), irrespective of any positive arguments for the principle. In presenting our arguments, we do however indirectly deal with any argument in favor of using the *de minimis* principle. For if we are correct, then arguments that aim to establish that the *de minimis* principle should be used in rational decision making must either be invalid or unsound.

We should also mention that to keep the discussion as focused as possible, we shall ignore an admittedly partly related literature on *risk acceptance* and *tolerability limits*. While the discussion of *de minimis* concerns which risks (if any) *to ignore*, the literature on risk acceptance and tolerability limits concerns which risks *to accept* (see, e.g., Aven & Renn, 2018, for a recent and very informative discussion). The reason for ignoring this literature is that although the concepts have clear similarities, the principles are also importantly different. For example, we may decide to accept the risk of side‐effects from some medicine, despite the risk being very high, if the expected benefit is sufficiently great. But that does not imply that the acceptable risk is *de minimis*. Moreover, one might argue that to accept a risk is in some sense to decide to ignore it after one has deliberated about what decision to make. However, if we apply the *de minimis* principle, we not only ignore a risk after the deliberation, but rather decide to remove it from consideration in the decision‐making deliberation (including the predecision risk analysis). Thus, although these concepts are similar, they are also relevantly distinct.

Another issue that might be worth clarifying before we proceed, is how we understand “risk”. When we use “risk” in this article, we mean the *probability* times the *severity* (i.e., undesirability or negative utility) of an unwanted outcome or event. However, Peterson (2002) uses “risk” in a different sense, referring to the probability component only. He does so to simplify and avoid an unnecessarily cumbersome terminology (see p. 48). Thus, in the upcoming discussion of Peterson's article, we make use of his notion of “risk”.

The concept of “probability” can also be understood in different ways (e.g., as subjective probabilities, frequencies, or propensities). But all of these have in common that a probability is a real number in the zero‐one interval that satisfies the standard (either finite or countable) additivity property, and is defined on an algebra of sentences or sets (for a discussion, see, e.g., Hájek, 2019). For the purpose of our argument, it makes no difference whether the *de minimis* threshold is defined with respect to subjective probabilities or objective ones (be they frequencies or propensities, say). If the threshold is defined for objective probabilities, then since a rational decisionmaker seeks to maximize expected value relative to her *estimate* (or subjective expectation) of objective probabilities,2For a classical discussion, see Lewis (1980). whenever these are available, such a decisionmaker will in many circumstances behave *as if* the threshold were defined relative to subjective probabilities. Therefore, the problems we raise below hold for such an agent, in those circumstances.

A final and related issue that might be worth clarifying, before we turn to our examination of *de minimis*, concerns the fact that two estimates that result in the same probability value might be based on very different types of knowledge—and, similarly, based on evidence of differing strength—and can correspondingly lead to different levels of certainty (or confidence) in the estimated probability value.3For extensive recent discussions about the importance of the knowledge base to risk analysis, see the articles in Aven and Zio (2018). To take a very simple example, if we on one occasion have the strongest possible evidence for a particular coin being unbiased, but on another occasion all we know about some other coin is that it is either double headed or double tailed, then on both occasions, our subjective probability for the coin coming up heads on its next toss might be 0.5. However, our “certainty” of this subjective probability—that is, our confidence in our estimate—is clearly much greater in the first case. Some people argue that a risk‐averse decisionmaker should show more caution the greater the uncertainty about the estimated probabilities (see, e.g., Stefánsson & Bradley, 2019). The implication of this view for *de minimis* could be that one should not apply the principle—or that one should use a lower threshold—when one lacks confidence in one's subjective probability. However, for the present purposes, we need not worry about the issue of uncertainty (thus understood). For whether the same *de minimis* threshold is supposed to hold for all levels of uncertainty, say, or only for some levels of uncertainty (or perhaps different thresholds for different levels), the problems that we raise will arise for each level of uncertainty for which a *de minimis* threshold is specified. Therefore, we will mostly ignore the level of uncertainty in this article.4An exception is the fifth section, where we simply illustrate why our argument holds irrespective of the level of (and independently of one's attitude to) uncertainty.


## 2 PETERSON'S ARGUMENTS AGAINST PREVIOUS FORMULATIONS

Peterson argues against three previous formulations of the *de minimis* principle: the specific‐number view, the nondetectability view, and the natural‐occurrence view. We briefly discuss these in reverse order.

*The natural‐occurrence view*: an (anthropogenic) risk R is *de minimis* provided the probability of R does not exceed the natural occurrence of this type of risk. (Peterson, 2002, p. 52; see ibid for more references)

As Peterson notes, the natural‐occurrence view is problematic for several reasons. First, there are clearly natural risks that are not *de minimis* (e.g., radon). Second, the view cannot deal with risks that do not occur naturally (p. 52f). Third, we may note that the view seems to violate what is called Hume's law, that is, that we cannot deduce *ought* from *is*. Fourth, it also seems to rest on the flawed *argument from naturalness*, according to which the natural is good and unnatural is bad. However, as Peterson points out, it is not always the case that the natural is good. Furthermore, even in situations when the natural actually is good, it is not so necessarily (cf. pp. 52–53; cf. also Takala, 2004, for a discussion on the argument from naturalness).

*The non‐detectability view*: a risk R is *de minimis* provided that it cannot be scientifically established (at a later point of time) whether R has in fact materialised or not. (Peterson, 2002, p. 50; see ibid for more references)

To counter the nondetectability view, Peterson uses an argument from Hansson (1999), which shows that the detectability of risks of equal probability depends on their previous distribution. Hansson considers exposure to three different substances—A, B, and C—all of which gives an absolute increase of the probability of death by 0.5%. In case of exposure to A, the risk increases from ∼0% to 0.5%; in the case of exposure to B, the risk increases from 1.0% to 1.5%; and in the case of exposure to C, the risk increases from 10.0% to 10.5%. Because of the statistical background conditions, we can identify the individual victims in A. However, in B, we can only statistically determine that 5 out of 15 people got the disease from exposure (but not identify who), and in C, the background conditions make it impossible to distinguish exposure from random variations. Yet, in all situations, the risk is the same, which arguably shows that the view is unreasonable.

*The specific‐number view*: a risk R is *de minimis* provided the probability of R falls below a certain number N (eg 10^−6^). (Peterson, 2002. P. 49; see ibid for more references)

As Peterson argues, the specific‐number view is arbitrary: it is hard to set a specific limit and suggestions of such limits have varied. Indeed, “[w]hy should we ignore a lifetime risk of 1.0 × 10^‐6^, but not one of 1.00001 × 10^−6^?” (p. 50). The idea of a specific limit seems to be a convention, not a basis for settling the limits of what is *de minimis*.

However, in light of his criticism, Peterson ends his article by suggesting an alternative so closely aligned with the *specific‐number view* that we can call it the *vague‐number view*. As the name indicates, Peterson argues that *de minimis* is a vague principle, meaning that while a *specific* number for the limit cannot be set, there is a *vague* limit. As Peterson argues, this can “explain why it has been so difficult to reach consensus about whether a lifetime risk of, say, 10^−6^ is negligible or not” (p. 53). However, that we cannot determine whether 10^−6^ is *de minimis* does not mean that the principle cannot be used. Indeed, on the standard analysis of vagueness, we can often determine whether a token is the extension of a vague concept or not. It is only when the limit of the concept is approached that this becomes indeterminable. Thus, according to Peterson, we can often distinguish between risks that are clearly *de minimis* and risks that are clearly *de manifestis*, even though there is no sharp limit between these concepts (p. 53).

We can formulate Peterson's view as follows:

- *The vague‐number view*: a risk R is *de minimis* provided the probability of R falls below a vague limit.

This is arguably a vague formulation of a vague principle, but part of the problem is that we do not know what this vague limit is. In the next section, we consider reasons to further specify Peterson's concluding (implicit) definition of *de minimis*, namely, that we must take the context into consideration. Next, we show that deciding upon the appropriate context will result in an application of the principle that is either normatively unacceptable or not feasible for boundedly rational agents. Lastly, we provide an argument against those versions of the *de minimis* principle according to which a *de minimis* risk can be completely ignored.

## 3 THE *DE MINIMIS* PRINCIPLE MUST BE CONTEXTUAL

According to Peterson, we can conclude that 10^−12^ is *de minimis*. But why is that? Should not an evaluation of whether any probability (or risk) is *de minims*, in part, depend on the context of the decision? As Fiksel notes, “Designation of a risk as *de minimis* is meaningful only in the context of the activity that produces it and the party that bears it” (1985, p. 258). However, the role of the context is not specified in the vague‐number view, which, we contend, means that it needs to be modified.

To illustrate that there is no general, context‐free, way to specify (vaguely or not) when a risk is *de minimis*, consider the fact that some have held that probabilities below 10^−4^ are *de minimis* (see Schrader‐Frechette, 1985, pp. 435–436). While this seems obviously erroneous, from our perspective, the proposal would seem much more reasonable if the global population were extremely small. Suppose, for instance, that the global population were 10^6^. If so, the idea of treating 10^−4^ as *de minimis* would seem less obviously erroneous.5The same argument holds if we focus not on probabilities, but on expected disutility.


On the one hand, imagine a small population of 100 people considering whether to treat a fatality risk with the probability of 10^−4^ over a lifetime as *de minimis*. If we suppose that *de minimis* is a sound principle, then we should think that it applies to such a situation. On the other hand, imagine that the world population is 10^20^. Would the presumption by Peterson that “no one seems to doubt that a lifetime risk of eg 10^−12^ is truly negligible” (p. 53) not seem a bit problematic?6Two anonymous reviewers for *Risk Analysis* point out that it seems unfair to Peterson to presume that the same probability limit applies to risks of large negative consequences as to risks of small negative consequences. However, our argument does not hinge on this interpretation of Peterson. In fact, the aim here is not to criticize Peterson. Rather, the aim is simply to show that if the *de minimis* principle is not formulated as a context‐sensitive principle, then it leads to absurd conclusions. (Indeed, Peterson may well have implicitly intended his principle to be context sensitive.) What we are suggesting here is that the context matters. In deciding about risks, we must take the so‐called *decisional horizon* into consideration (i.e., we must decide what to include and exclude in the risk evaluation; cf. Toda, 1976). But this also means—as the two examples above show—that there is no (vague, or nonvague) limit that we can set as *de minimis* in all contexts.

Proponents of the *de minimis* principle may respond and say that this is, in fact, because *de minimis* is contextually sensitive (i.e., beyond the fact that Peterson may be right to claim that the principle is vague, the context also matters). Thus, we cannot set a general limit because we cannot determine whether a risk is *de minimis* without taking the context into consideration. It is only in relation to such a context that a risk is *de minimis*.

Hence, let us reformulate the principle:

- *The contextually sensitive vague‐number view*: a risk R is *de minimis*, in a given context, provided the probability of R falls below a vague limit, determined by the context.

## 4 THE CONTEXTUAL *DE MINIMIS* PRINCIPLE IS UNACCEPTABLE

In the previous section, we argued that the *de minimis* principle must be context sensitive; otherwise, the principle is simply too implausible. The problem, however, is that if we accept a context‐sensitive *de minimis* principle, such as the contextually sensitive vague‐number view, then the application of the principle will give contradictory results depending on the perspective of a decisionmaker. To see this, suppose that ≤10^−n^ is *de minimis* if (and only if) applied in a context such that a risk R is imposed on ≤*m* people. First, imagine that a population, *C*, of >*m* people are considering whether a risk *R* ≤ 10^−^ *^n^* is acceptable. Since *C* has >*m* people, *R* is not *de minimis* in the given context. Next, imagine that the evaluation is done on more local level, and that for each local level, *L*_1_…*L_n_*, *R* ≤ 10^−^ *^n^* would be imposed on ≤*m* people. If so, *R* would be *de minimis* in each local context.

Thus, R can both be *de minimis* and not *de minimis*, depending on the context. This, of course, follows directly from the contextual nature of the contextually sensitive vague‐number view. Indeed, this is arguably a feature, not a bug, since it recognizes that the evaluation of the risk R *should* vary between contexts. However, this only pushes the hard question to another level: Should the evaluation be done on *C* as a whole or in *L*_1_, …, *L_n_* individually?

Suppose that if the decision is made on the local level, then the risk will be considered acceptable. But if the decision is made on the level of the whole population extended over time, the risk will not be considered acceptable. Suppose, further, that if the people of *C* (i.e., all the people *L*_1_…*L_n_*) are exposed to the risk, then there is close to 100% chance that one person will die.

Assuming that one person's sure death cannot be *de minimis*, it seems that the decision should be made on the whole population level, not on the local level. But unfortunately, it will generally not be possible, in particular for boundedly rational people, to apply *de minimis* at the whole population level. To take an example, a regulator who is considering whether some particular risk imposed by a new chemical is *de minimis*, needs to consider not only the risk that the chemical *in isolation* imposes on the whole *current* population, but also the risk imposed in the long run (cf. Mumpower, 1986), given what actions are expected to be taken (e.g., about the introduction of other chemicals). Obviously, such an evaluation will not be possible for a bounded agent (nor, perhaps, for ideally rational agents, since the future may not be fully determined).

Thus, either the principle should be applied at localized levels, in which case it will result in total evaluations that clearly go against the spirit of the principle (e.g., by treating a sure death as being *de minimis*); or it should be applied to a whole population over time, in which case it will not be applicable by boundedly rational agents, or in fact by perfectly rational agents with imperfect prediction powers. If the latter (“global approach”) is how one should apply a *de minimis* principle, then that seems to show that, contrary to what Adler (2007) suggests,7In fairness to Adler, we should stress that he only claims that the reduction of decision costs is (given, e.g., welfare consequentialism) a *potential* justification for *some* version of the *de minimis* principle, but he is quick to note that no justification has been provided for any *particular* version of the principle. the *de minimis* principle (properly applied) does not reduce decision costs. The trouble with the former (“local”) approach, however, is that the very idea behind *de minimis* seems to be to allow decisionmakers to ignore (or at least pay little attention to) risks that we can be pretty sure will not make any difference. But a sure death surely does make a difference. Lastly, if we assume that the decisionmaker is completely free to decide whether the principle should be applied locally or globally, then the principle will result in contradictory recommendations, depending on whether the local or the global perspective is adopted.

## 5 COMPLETELY IGNORING *DE MINIMIS* IS UNACCEPTABLE

Finally, we want to mention an objection that only applies to some members of the family of *de minimis* decision principles, namely, those that say that risks below some threshold can be completely ignored. Suppose that we apply such a principle to a decision between options A and B. The options differ only in that A has *no risk* and B has a *de minimis* risk; otherwise, the two options have the same benefits and the same costs, etc. We moreover assume that we have equally strong evidence for the claim that A has no risk while B has a *de minimis* risk, that is, the level of uncertainty is the same when evaluating these two options. To make the example as simple as possible, we can even assume that we are *certain* that A has no risk and that we are also *certain* that B has a *de minimis* risk. Now, the versions of the *de minimis* principle under discussion would suggest that B is just as good as A. But this is obviously not true. Indeed, A is superior to B—and a decision principle that suggests otherwise should be jettisoned.

To further illustrate the above point, suppose that we can describe the two options (A and B) in the following matrix, where s1 to s3 denote possible *states of the world*, and the numbers in the cells ordinally represent the desirability of the various possible outcomes of the two options (which means that the numbers only denote the outcomes’ relative ranking).

**Table:** 

	s1	s2	s3
A	1	1	1
B	1	1	−1

Now, if the probability of s3 is below the stipulated threshold, that is, if the risk of −1 is *de minimis*, then A and B are equally good, according to the versions of *de minimis* under discussion.8The argument of course also holds if the expected disutility of the outcome of B in s3 is below some stipulated utility threshold. And, recall that if we think that *de minimis* principles should be sensitive to the level of uncertainty about probability estimates (as described in the Introduction), then we can interpret the example such that the level of uncertainty is one for which some *de minimis* principle is taken to be applicable. But that means that this type of *de minimis* principle violates *State‐Wise Dominance* (and thus also *Stochastic Dominance*, since the latter entails *State‐Wise Dominance*), which implies that since the outcome of A is at least as good as the outcome of B in any state of the world, and the outcome of A is moreover strictly better than the outcome of B in some state of the world (s3), A should be preferred to B. No good rule for handling risk should violate state‐wise dominance.

A proponent of *de minimis* may respond that the above is a highly idealized example with limited (if any) practical relevance. In real life—the proponent could further argue—we can never know with certainty that one alternative state‐wise dominates the other.9We thank an anonymous reviewer for *Risk Analysis* for bringing to our attention the need to respond to this objection. So, let us make the example concrete to illustrate that it need not, in fact, be esoteric or unrealistic.

Imagine that a risk analyst is preparing to propose alternatives to a committee that decides between different types of paint on a playground. The risk analyst is instructed to rank different types of paint according to a very narrow criterion, namely, only in terms of the health risks that the different types impose (such as whether they are cancerogenic). Now, it does not seem implausible that, given the limited criterion according to which the risk analyst ranks the different types of paint, one type of paint could state‐wise dominate another. For example, it seems plausible that one type of paint could include a chemical that is known to increases the risk of cancer, by some extremely small probability *n*, while another type of paint is taken not to be cancerogenic. Given *de minimis*, it would follow, for some such *n*, that the risk of cancer that the first type of paint has would be *de minimis*. To take an example, if we suppose that the paint can cause cancer in children who have some very rare condition, then s3 can be interpreted as the possibility that a child with this condition enters the playground in question. Moreover, it seems plausible that, according to the narrow criterion that the risk analyst is instructed to use, these two types of paint are otherwise identical. But then a structure like that illustrated by the above matrix may well arise, in which case one alternative state‐wise dominates another, even though they will be considered equal if the *de minimis* principles under discussion is part of the risk analysis.

Furthermore, the relevance of the example can also be illustrated by extending the argument in various other ways. For example, the *de minimis* principle seems to indicate that safety measures for *de minimis* risk should not be used. However, that a risk is *de minimis* does not necessarily imply that safety measures are not cost‐efficient (Mumpower, 1986). Indeed, the principle even suggests that low‐cost (or even cost‐free) safety measures can be ignored if the risk is sufficiently small—which seems as erroneous as claiming that the choice between A and B is indifferent. In sum, the fact that the (strong version of the) *de minimis* principle gives such demonstrably wrong guidance in some cases is, we believe, sufficient reason to reject (this strong version of) the principle.

## 6 CONCLUSION

We conclude that *de minimis* reasoning has no place in rational decision making. There is no probability threshold below which risks can rationally be treated categorically differently from other risks. Moreover, from the perspective of bounded rationality and decision making with limited information, it is unclear whether *de minimis* principles are of any use. There are strong reasons to think that we must only apply such principles to what we might call “global” decision problems—for example, those involving a whole population or a whole economy, both extended in time—rather than local ones—for example, those involving small subpopulations or subsectors of the economy at a time. But such global decision problems are clearly not solvable by ordinary (bounded and information constrained) agents (for a discussion, see Stefánsson, 2019).
